# Machine learning to refine decision making within a syndromic surveillance service

**DOI:** 10.1186/s12889-019-6916-9

**Published:** 2019-05-14

**Authors:** I. R. Lake, F. J. Colón-González, G. C. Barker, R. A. Morbey, G. E. Smith, A. J. Elliot

**Affiliations:** 1 School of Environmental Sciences, University of East Anglia, Norwich, NR4 7TJ UK; 2 National Institute for Health Research Health Protection Research Unit in Emergency Preparedness and Response, London, UK; 3 Real-time Syndromic Surveillance Team, Field Service, National Infection Service, Public Health England, Birmingham, B3 2PW UK

**Keywords:** Syndromic surveillance, Public health, Decision making, Bayes’ theorem, Machine learning, Artificial intelligence

## Abstract

**Background:**

Worldwide, syndromic surveillance is increasingly used for improved and timely situational awareness and early identification of public health threats. Syndromic data streams are fed into detection algorithms, which produce statistical alarms highlighting potential activity of public health importance. All alarms must be assessed to confirm whether they are of public health importance. In England, approximately 100 alarms are generated daily and, although their analysis is formalised through a risk assessment process, the process requires notable time, training, and maintenance of an expertise base to determine which alarms are of public health importance. The process is made more complicated by the observation that only 0.1% of statistical alarms are deemed to be of public health importance. Therefore, the aims of this study were to evaluate machine learning as a tool for computer-assisted human decision-making when assessing statistical alarms.

**Methods:**

A record of the risk assessment process was obtained from Public Health England for all 67,505 statistical alarms between August 2013 and October 2015. This record contained information on the characteristics of the alarm (e.g. size, location). We used three Bayesian classifiers- naïve Bayes, tree-augmented naïve Bayes and Multinets - to examine the risk assessment record in England with respect to the final ‘Decision’ outcome made by an epidemiologist of ‘Alert’, ‘Monitor’ or ‘No-action’. Two further classifications based upon tree-augmented naïve Bayes and Multinets were implemented to account for the predominance of ‘No-action’ outcomes.

**Results:**

The attributes of each individual risk assessment were linked to the final decision made by an epidemiologist, providing confidence in the current process. The naïve Bayesian classifier performed best, correctly classifying 51.5% of ‘Alert’ outcomes. If the ‘Alert’ and ‘Monitor’ actions are combined then performance increases to 82.6% correctly classified. We demonstrate how a decision support system based upon a naïve Bayes classifier could be operationalised within an operational syndromic surveillance system.

**Conclusions:**

Within syndromic surveillance systems, machine learning techniques have the potential to make risk assessment following statistical alarms more automated, robust, and rigorous. However, our results also highlight the importance of specialist human input to the process.

## Background

In many countries, the automatic recording of healthcare seeking behaviour for public health surveillance is increasingly efficient and sophisticated. For example, such recording may include calls to medical telephone helplines and emergency department attendances [1]. These developments in recording techniques have been possible as a result of improved patient management systems but they also satisfy a desire for improved and early identification of potential public health threats. The developments are also important for providing reassurance of the absence of a threat and, more generally, reflect increased preparedness in relation to public health issues or emergencies [2]. Collection and analysis of recorded healthcare seeking behaviour, in the form of symptoms/syndromes rather than confirmed diagnoses, is more timely than other traditional surveillance schemes for monitoring public health such as laboratory reporting, and is described as ‘syndromic surveillance’ [3]. The collected information and subsequent analytical processes involved in syndromic surveillance provides a complex statistical picture that represents both the actual incidence of ill-health within a population and the pattern of healthcare seeking behaviour [4].

In England, the ‘rising activity, multi-level mixed effects, indicator emphasis’ (RAMMIE) method for anomaly detection is used to generate statistical ‘alarms’ from syndromic surveillance data [5]. Similar statistical aberration detection systems exist elsewhere (e.g. Farrington; [6], Early Aberration Reporting System; [7]). Syndromic surveillance systems have to follow up alarms with a second stage process to determine whether individual alarms are of potential public health importance and thus need to be communicated to public health colleagues. However, our experience of other worldwide systems in operation is that these processes are often based upon expert opinion and largely undocumented. The syndromic surveillance service in England is distinctive due to its size and complexity. It analyses data from four different surveillance systems, each of which are composed of multiple data sources. The English system has led to the development of a formal risk assessment process to standardise the second stage processes involved in deciding which statistical alarms are of potential public health importance [8].

The English risk assessment process comprises an initial assessment of the epidemiological data underlying the alarm based upon several questions, such as the size of alarm. Each question is scored and in the vast majority of cases if the final score exceeds a specified value, a second stage is performed. The second stage involves a review of the data by a consultant epidemiologist, incorporating further epidemiological data. Based on the total scores from stage one and stage two, and considering all other available evidence, a decision is made. The decision can be to initiate an ‘Alert’ identifying the alarm as of potential public health importance. Alternatively, the decision can be to continue to ‘Monitor’, or simply assign ‘No-action’ to the statistical alarm (Further detail in [8]).

Automated time-series analysis of actual healthcare seeking behaviour needs to avoid false negatives, and so it produces a high proportion of alarms that, on consideration, require no further action. To achieve this analysis, the risk assessment process used in England requires notable investments in terms of syndromic surveillance analyst time, training, and maintenance of an expertise base. In England, around 100 statistical alarms are generated every day but only one in every thousand is identified as requiring public health action. Only six in every thousand alarms are identified as requiring further monitoring. These rates make data interpretation challenging from a human perspective as the risk assessment process has to consider an information supply in which events that require public health action or monitoring are embedded within a much larger volume of events which do not require action or further monitoring.

Therefore, the aims of this study were to evaluate machine learning as a tool for computer-assisted human decision-making when assessing statistical alarms. Specifically we explore whether machine learning techniques for multi-state classification (i.e. ‘No-action’, ‘Monitor’, ‘Alert’), can refine the decision making process. Such an investigation is unique from a syndromic surveillance perspective and challenging from a computational perspective due to the very small proportion of alarms which are classified as ‘Alerts’. This imbalance, usually called asymmetric data, makes the interpretation of statistical variations problematic.

In this paper, we use machine learning techniques to build a classifier that can support the existing risk assessment process. We have concentrated on Bayesian classifiers, where the assignment of outcomes is based on probabilities learned from the data set. Other approaches - such as the C4.5 classifier defined by Quinlan [9], support vector machines and random-forests - are equally relevant. Bayesian networks are chosen because they give particularly intuitive results and they are useful when implementing machine learning in a new context, in this case syndromic surveillance. Here, three Bayesian classifiers are applied to the Decision record from the risk assessment process. Two further Bayesian classifiers were implemented to account for the predominance of ‘No-action’ outcomes in the risk assessment. The results from each classifier are fully evaluated using a range of classification performance metrics.

## Methods

### Syndromic surveillance data

Public Health England (PHE) coordinates a national syndromic surveillance service based around four real-time syndromic surveillance systems. The first two systems are based on records of consultations with medical doctors known as General Practitioners (GP). Data are obtained for out-of-hours consultations (GP out-of-hours syndromic surveillance system; GPOOHSS) and in-hours consultations (GP in-hours syndromic surveillance system; GPIHSS). Syndromic data are also obtained from a sentinel network of emergency departments (EDSSS) and the National Health Service telephone advice system (NHS111) [1, 10]. In this study we additionally included a very small number of events from the NHS Scotland telephone advice system (NHS24) to which PHE contributed in the past [11]. Anonymized data from these syndromic surveillance systems are aggregated to daily totals to produce time-series for many syndromic indicators, with aggregation at multiple levels of geographical resolution (e.g. regional totals per day) and age bands. Every day the RAMMIE statistical aberration detection system analyses more than twelve thousand separate time-series. Two types of alarms are generated indicating whether the activity is unusual given the time of year (historical alarm) or whether there has been a recent increase in activity (spike alarm) [5].

### Data pre-processing

PHE maintains a database of the risk assessment decision making process for audit purposes, which it made available to our research. The database covered a continuous period between August 2013 and October 2015 and contains details of 67,505 statistical alarms with the corresponding decision outcomes. This database provided the training data set for this study [8]. These data were systematically pre-processed. This pre-processing was completed manually using systematic tools in a text editor to correct spellings, remove duplicate records etc.

Table 1 lists the fields of these data, the permissible values and statistics on field completeness. Further pre-processing of the training data set, as summarized below, generated several of these fields. In Table 1 we are trying to determine the “Decision” which is whether at the end of the risk assessment process the outcome was ‘Alert’, ‘Monitor’ or ‘No-action’. There are 25 attribute variables that are used to determine the Decision. In Table 1, these 25 attributes are listed and categorized into those from the inherent features of the event, followed by those from the two stages of risk assessment.

**Table 1:** Attributes included in the development of a classifier for statistical alarms recorded by a PHE multi-system syndromic surveillance service

Field Name	Description	Entries	Missing	Unique	I_p_	Values	*p*-value
*Class Variables*							
*Decision*	Decision taken by syndromic surveillance analyst	592	66,913	3	1.0000	Alert, Monitor, No-action	–
*Attribute Variables; from event*							
*Year*	Year of the alarm	67,505	0	3	0.0001	2013, 2014, 2015	9.7 × 10^−2^
*Q*	Quarter	67,505	0	4	0.0002	Jan-Mar, Apr-Jun, Jul-Sep, Oct-Dec	3.3 × 10^−2^
*D*	Day of the week	67,505	0	7	0.0006	Sun, Mon, Tue, Wed, Thu, Fri, Sat	6.9 × 10^−8^
*Alarm*	Was the event a statistical alarm?	67,505	0	3	0.0014	Yes, No, Unknown	< 10^−10^
*System*	The system that alarmed	67,505	0	5	0.0006	NHS111, NHS24, EDSSS, GPOOHSS, or GPIHSS	4.4 × 10^−9^
*IndicatorS*	Indicator that alarmed	67,505	0	53	0.0041	1 of 53 different syndromes	< 10^− 10^
*IndicatorG*	Coarse grained version of *IndicatorS*	67,505	0	8	0.0013	Cardiac, Impact of Cold, Gastrointestinal, Impact of Heat, Influenza-like illness, Respiratory, Other & Unspecified	< 10^− 10^
*IndicatorP*	Specific/general indicator	67,505	0	2	0.0001	specific, General	2.0 × 10^−3^
*IndicatorL*	Indicator severity	67,505	0	5	0.0002	Consultation, Admitted, Severe, High Dependency Unit/Intensive Care Unit, Mortality	1.0 × 10^−10^
*Region*	PHE Region	67,505	0	13	0.0037	1 of 13 PHE regions	< 10^−10^
*LocationP*	Geography of alarm	67,505	0	3	0.0037	Local, Regional, National	< 10^−10^
*Experience*	Is syndromic surveillance analyst experienced?	67,505	0	2	0.0001	Yes, No	2.1 × 10^−2^
*Attribute Variables; from first stage risk assessment*							
*Excess*	Size of the alarm	66,406	1099	4	0.0115	0,1,2,3	< 10^−10^
*Repeated*	Is the alarm a repeat?	65,766	1739	4	0.0026	0, 1,2,3	< 10^−10^
*Multi-system*	Is the alarm in multiple systems simultaneously?	65,742	1763	4	0.0094	0,1,2,3	< 10^−10^
*Nattrend*	Is the alarm counter to the national trend?	65,771	1734	4	0.0003	0,1,2,3	2.3 × 10^−5^
*Score1*	Sum of scores from first stage risk assessment	65,795	1710	13	0.0277	0–12	< 10^−10^
*BInitial*	Does first stage analyst engage consultant epidemiologist to perform second stage?	67,505	0	2	0.0357	Yes, No	< 10^−10^
*Attribute Variables; from second stage risk assessment*							
*Season*	Is the alarm counter to the seasonal trend?	573	66,932	3	0.0258	Yes, No, Missing	< 10^−10^
*Geography*	Does the alarm show an atypical geographical clustering?	572	66,933	3	0.0259	Yes, No, Missing	< 10^−10^
*Age*	Is the alarm centred on a particular age group?	572	66,933	3	0.0264	Yes, No, Missing	< 10^−10^
*Severity*	Is there an unusual increase in illness severity associated with the alarm?	571	66,934	3	0.0259	Yes, No, Missing	< 10^−10^
*BScore*	Are the second stage scores subsequently completed?	67,505	0	2	0.0130	Yes, No	< 10^−10^
*Score2*	Sum of scores from second stage risk assessment	67,505	0	15	0.0325	1–15	< 10^−10^
*Bsummary*	Presence of text in summary field	67,505	0	2	0.0041	Yes, no	< 10^−10^

Notes: I_p_ is the amount of information obtained about the decision through observing the attribute (the mutual information between an attribute and decision)

*P*-value is a significance obtained from a Pearson χ^2^ measure of the association between a variable and the Decision

### Data: inherent features of event

The first attribute was the acquisition date which was partitioned into three fields representing the year, the quarter (Jan-Mar, Apr-Jun, Jul-Sep, Oct-Dec) and the day of the week (labelled “Year”, “Q” and “D”). The next attribute was “Alarm” which indicates whether the event produced a statistical alarm. Although the vast majority of events in these data were statistical alarms, a small fraction (~ 0.1%) were added manually by an analyst based on other surveillance observations. These manual additions may be a visually observed change in syndromic activity which was thought noteworthy but did not lead to a statistical alarm.

Each event can be identified by several elements. The attribute “System” encodes the syndromic surveillance system that is the source of the record (i.e. NHS111, NHS24, EDSSS, GPOOHSS, or GPIHSS). The next identification is the syndromic indicator that alarms. This identification is “IndicatorS” which can take on 53 possible values, reflecting the wide range of conditions that are monitored and the different categories of symptoms used between syndromic systems (e.g. diarrhoea, asthma, fever). Coding is not directly comparable between systems. Hence, a child with influenza could be classed as a fever on NHS 111 but influenza-like illness on GPIHSS [12]. For simplicity, we also mapped the 53 possible values of “IndicatorS” onto a coarse-grained attribute “IndicatorG” with only 8 categories. The indicator attribute is further partitioned into “IndicatorP” which captures whether the indicator is general or specific. “IndicatorL” specifies EDSSS events that reflect severity ranging from a standard consultation to a High Dependency Unit/Intensive Care Unit. Grouping the indicators in this way helps to reduce noise in the data and the possibility of overfitting.

Almost 250 geographical locations are identified in the training data set and these are mapped onto one of 13 PHE regions (“Region”). The geographical scale of location information (i.e. local, regional or national) is encoded with “LocationP”. Finally, the training data set identifies the syndromic surveillance analyst as one of 15 different individuals. This identification was used to establish the experience of the analyst which is coded as a binary variable called “Experience”. In principle “Experience” could capture the influence of new analysts within the decision making process. An experienced analyst was classified as an individual who had undertaken the risk assessment process for more than 5% of the previous 2500 assessments.

### Data: stages 1 and 2 of the risk assessment

During the risk assessment, the attributes of each event are interpreted by the syndromic surveillance analyst (Table 1 and [8]). In stage 1 four attributes are scored: the size of the “Excess” recorded by syndromic data; whether the alarm is a recent “Repeat”; whether it is counter to the national trend “Nattrend”; and whether it is signalled by multiple systems “Multi-system”. The “Excess” and “Nattrend” scores are based upon heuristic judgements. The four first round scores are summed to provide “Score1”. Using this score and any additional unrecorded information, such as consultations with field epidemiologists, the analyst decides on whether stage 2 of the assessment, where a consultant epidemiologist is engaged, should occur. This consequence is captured in a field “Binitial”.

If stage 2 occurs, four more scores are added to the data set. These scores indicate whether the alarm is counter to a seasonal trend (“Season”), whether there is an atypical geographical clustering (“Geography”), whether the event is centred on a particular age group (“Age”) and whether there is an unusual increase in illness severity (“Severity”). These four scores are added to Score1 to create “Score2”. An attribute “BScore” indicates whether the second stage scores are completed. In addition, the risk assessment record contains a free text field where the consultant epidemiologist can provide a summary of each event. The free text field has not been interpreted but an attribute has been created - “BSummary” - recording the presence or absence of information in this field. Approximately a third of the recorded events contain such information.

The data set includes many missing values so in the decision outcome field the absence of an outcome is interpreted as ‘No-action’. The first stage scores are sometimes left blank, possibly when the expert has jumped to an immediate decision choice. Where this happens, the corresponding scores were all assigned to zero. The same recoding was performed in the second stage analysis. In the analysis, all scores and totals are considered as labels rather than as numerical values.

Higher overall scores are more likely to correspond with the alert outcome, but this mapping is not deterministic and some low scoring alarms may also lead to an alert. The average first stage score for alarms that generate an alert is higher than that for alarms that generate ‘No-action’ (6.81 vs 5.04, *p* = 1.8 × 10^− 8^).

### Method: Naïve Bayes (NB)

**Fig. 1:**
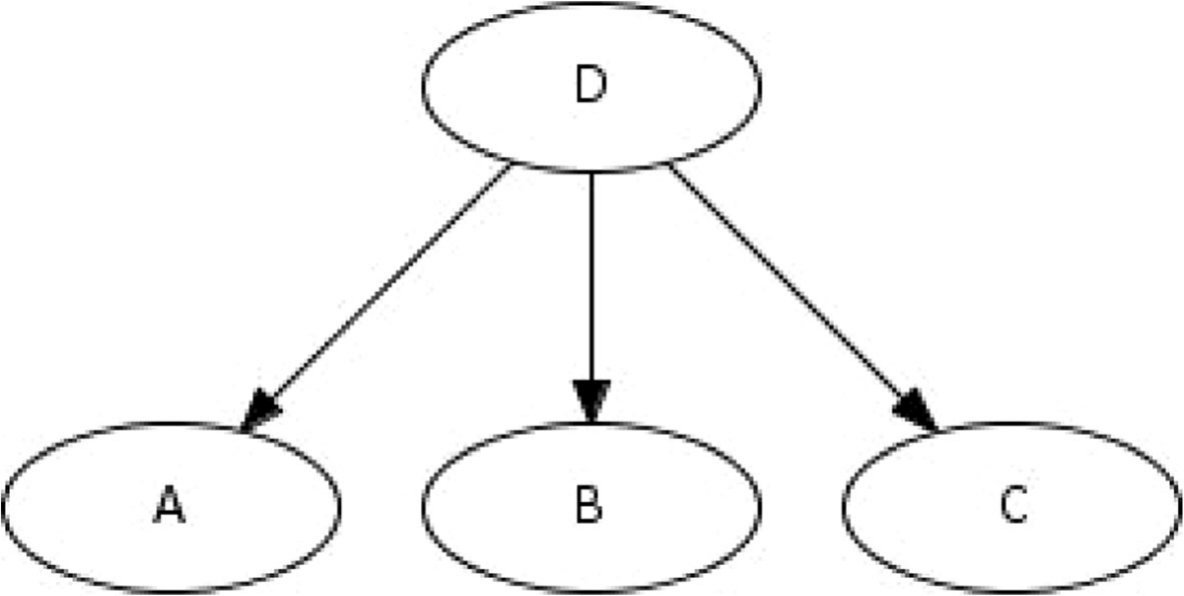
A simple 4 node network representing the conditional independence of observed symptoms A, B and C given a patient with disease D and a representation of naïve Bayes decision making

The naïve Bayes (NB) classifier is a special type of probabilistic graphical models known as Bayesian networks. NB is the simplest, and often most effective, Bayesian classifier [13]. NB assumes that the state of an attribute depends on the decision outcome but, given this information, it is conditionally independent from all the other attributes. An example of NB can be represented by the connection between the presence of an event leading to an alert D and observations of three binary attributes A, B and C. NB assumes that if the event is an alert, the probability of observing attribute A does not depend on attribute B (Fig. 1). Thus, NB says that if the event is classified as an alert D the probability that A is true or false does not depend on whether B is true or false. The independence assumption means that the very complex probability that describes whether the event is an alert D - given observations for all three attributes, A, B and C - is easy to calculate using Bayes’ theorem. The calculation can be represented by a simple fan-like node and arrow diagram such as in Fig. 1, where the arrows represent a “depends on” relationship between nodes. For example, the presence or absence of attribute A depends on the presence or absence of the alert D, but not on the values of the other two attributes.

Thus, if the class of an individual event is known, it is possible to assign probabilities to the states of each attribute without knowing the state of other attributes. For example, if the Decision is ‘Alert’ it is possible to assign the probability that “System” is ‘EDSSS’ without knowing the condition (i.e. that “IndicatorS” is ‘Gastrointestinal’).

In terms of probability the NB structure means that the complex joint probability of the attributes can be expressed as a simple product, i.e. *p(A*_*1*_*, A*_*2*_*, … A*_*N*_*| C) = p(A*_*1*_*| C) x p(A*_*2*_*| C) x … x p(A*_*N*_*| C)* where *A*_*i*_ are *N* attributes and *C* is the class variable. Further, each of the component probabilities on the right can be estimated from the data set by counting frequencies of the states of each attribute. For example when the “Decision” is ‘Monitor’ how often is the “System” equal to ‘GPOOHSS’. Crucially, Bayes’ theorem then connects the conditional probability for the attributes with the opposite conditional probability that determines classification - *P(C | A*_*1*_*, A*_*2*_*, .. A*_*N*_*)* - so that within the naïve Bayes’ approximation the global classification problem is a relatively simple computational step. This computation is easy to perform manually but is also supported in many sophisticated Bayesian Network software tools, such as Hugin (Hugin Expert A/S, Aalborg). The learning is initiated with uniform prior frequencies [14], which do not have a significant influence on the results. Each case in the database then adds one to the frequency count in the learning process.

### Method: tree-augmented Naïve Bayes (TAN)

Natural extensions for the NB classifier address the possibility that some of the attribute variables may be dependent on each other given the class variable. In such a situation, returning to Fig. 1, the presence or absence of attributes A and B may depend on each other as well as the presence or absence of an alert D. NB neglects these dependencies and may therefore include some double counting of evidence leading to misclassification [15]. One solution to this situation is to augment the structure of the network by adding links between attributes. This structure accommodates the additional between-attribute dependencies that are captured in the data set. Discovering optimal feature sets or optimal augmentation is computationally complex but, with some restrictions placed on the possible set of additional links, it is practical to induce valuable augmented network classifiers from data. This latter option is implemented in this paper through TAN where additional links are created between attributes, constrained only within a tree structure, i.e. each attribute has a maximum of two parents including the class variable and one other attribute. The best set of tree-augmenting links can be discovered with the Chow-Liu algorithm [16]. Corresponding conditional probability tables can be established from the training data set using an expectation-maximization approach that is often used for learning Bayesian networks [17].

### Method: Bayesian multinets (multinet)

TAN represent dependency among the attributes in agreement with that captured in the complete training data set. As a technique TAN assumes that the dependency structure is independent of the state of the class variable. This assumption means that for a strongly asymmetric data set, such as the one used here, the dependency of attributes will be mainly the result of information in ‘No-action’ events. However, from a public health perspective we are more interested in ‘Alerts’ and ‘Monitor’.

Bayesian Multinets are a generalization of TAN that allow the relations between the features to be different for different values of the class node [18]. Thus, for each Decision separate networks are produced with different structures, unlike TAN where the relations between attributes are the same for each class attribute.

### Method: weighted networks (TAN* and multinet*)

Large asymmetry in the data source, in this case the predominance of ‘No-action’, has a major impact on classifier construction and performance, particularly in relation to classification of the rare outcomes. For probabilistic tools the classification process usually involves identification of the output class that has the highest probability, but alternative decision steps can be used to account for the data asymmetry. In a modified TAN approach (TAN*) outcomes were classed as ‘Alert’ if the ‘Alert’ probability was *≥0.2*. If not, they were classified as ‘Monitor’ if the ‘Monitor’ probability was *≥0.2*. If neither of these two thresholds were met then the Decision was classified as ‘No-action’.

A variety of weighted learning procedures, which either oversample the minority class or undersample the majority class during the learning step, have been developed to address the asymmetry issue [19, 20]. It is possible to mimic the oversampling approach by modifying the prior probabilities placed on the decision outcomes in the Multinet construction. In our case, this amounts to oversampling the minority class (i.e. Decision = ‘Alert’) by approximately 1000. This is implemented for the Multinet classifier (Multinet*).

### Method: evaluating classifier performance

Classifier performance can be estimated using ten-fold cross validation [21]. This method involves splitting the training data set into ten random subsets of approximately equal size. The first subset is used as a validation set, and the remaining nine subsets are combined as a training set for constructing the classifier. The process is repeated with each subset acting as the validating set, enabling the trained classifier to predict each of the three outcomes ‘Alert’, ‘Monitor’, and ‘No-action’ on the validation set. These results are compared with the actual decisions recorded in the validation set. Cross validation provides a prediction for each event in the full data set. A confusion matrix is then produced as a table that shows how events with a particular Decision outcome in the full training data are assigned to a particular Decision outcome by the classifier. A perfect classifier would have a confusion matrix where the number of misclassifications was zero. Four measures of performance are calculated, with each measure described in Table 2 [23].

**Table 2:** Classification performance measures

Measure	Description
accuracy	Proportion of correct predictions made by the classifier.
Matthews correlation coefficient (MCC)	Calculated for each outcome separately. Varies between − 1 and 1, and is similar to a Pearson correlation. It is evaluated from all the elements of the confusion matrix. Gives a more balanced quantification of performance than accuracy as it considers how closely the predicted results follow the decisions in the test data. Other correlation measures exist, but the MCC is suited to asymmetric classes and multi-state systems [22].
Precision (positive predictive power)	Calculated for each outcome separately. Expresses the fraction of classifications that match the true outcome. True positives/(true positives + false positives). E.g. proportion of ‘Alerts’ produced by the classifier that were ‘Alerts’ in the risk assessment database.
Recall (sensitivity)	Calculated for each outcome separately. Expresses the proportion of each outcome that is correctly returned by the classifier. True positives/(true positives + false negatives). E.g. proportion of ‘Alerts’ in the risk assessment database that were identified by the classifier.

## Results

The performance of each of the five classifiers is presented as a confusion matrix in Table 3. The NB classifier has an accuracy of 99.1% indicating that 99.1% of the produced classifications were correct. Accuracy values for TAN, TAN*, Multinet and Multinet* are 99.5, 99.3, 99.4, and 98.5% respectively. However, from a public health perspective accuracy is a poor indicator of performance as it is dominated by the high proportion of ‘No-action’ Decisions. The Decisions ‘Monitor’ and in particular ‘Alert’ are most important from a public health standpoint.

**Table 3:** Confusion matrix for classification of statistical alarms recorded by a multi-system syndromic surveillance service in England

			Classification		
			Alert	Monitor	No-action
Decision	NB	Alert	32	22	8
		Monitor	42	269	69
		No-action	30	469	66,564
	TAN	Alert	21	27	14
		Monitor	12	227	141
		No-action	17	144	66,902
	TAN*	Alert	27	24	11
		Monitor	22	260	98
		No-action	27	293	66,743
	Multinet	Alert	11	19	32
		Monitor	1	173	206
		No-action	1	138	66,924
	Multinet*	Alert	24	36	2
		Monitor	8	315	57
		No-action	17	866	66,180

*Modified approach to account for data asymmetry i.e the predominance of ‘No-Action’ outcomes

Table 4 presents further performance measures for each Decision separately, focussing upon ‘Alert’ and ‘Monitor’ which are most important from a public health standpoint. We additionally provide metrics for the Decision ‘Alert’ *or* ‘Monitor’, as they both require some kind of action. These metrics were generated by merging the ‘Alert’ and ‘Monitor’ data from Table 3. They are therefore not completely consistent as the classifier probabilities reflect three possible outcomes.

**Table 4:** Performance measures (tenfold cross validation) for multi-state classification of statistical alarms in the PHE multi-system syndromic surveillance service

			NB	TAN	TAN*	Multinet	Multinet*
Decision	Alert	MCC	0.398	0.377	0.393	0.387	0.435
		Precision	0.308	0.420	0.355	0.846	0.490
		Recall	0.516	0.339	0.435	0.177	0.387
	Monitor	MCC	0.497	0.581	0.552	0.486	0.459
		Precision	0.354	0.570	0.450	0.524	0.259
		Recall	0.708	0.597	0.684	0.455	0.829
	Alert + Monitor (merging decisions/classifications from Table 3)	MCC	0.587	0.643	0.617	0.521	0.507
		Precision	0.422	0.641	0.510	0.595	0.303
		Recall	0.826	0.649	0.754	0.462	0.867

*Modified approach to account for data asymmetry i.e the predominance of ‘No-Action’ outcomes

Table 4 indicates that for the Decision ‘Alert’, the NB classifier has an MCC, similar to a Pearson correlation, of 0.398. The precision for ‘Alert’ outcomes are 0.308 indicating that 30.8% of ‘Alert’ outcomes produced by the NB classifier were similarly classified in the risk assessment database. The recall for ‘Alert’ was 0.516 indicating that 51.6% of ‘Alert’ outcomes in the risk assessment database were similarly classified by the NB classifier. A similar pattern emerges for the ‘Monitor’ Decision, but the metrics are uniformly higher, with a notable jump in recall over precision (0.708 vs 0.354). For public health decision making, sometimes a high recall is preferred to a high precision. A preference for high recall is because the purpose of the system is outbreak detection, and systems are designed to deal with a modest burden of investigation even if some of the alarms investigated are assessed as false positives. For the NB classifier, when ‘Alert’ and ‘Monitor’ outcomes are merged there is an increase in MCC, precision and recall over their constituents. For recall, the value is 0.826 indicating that 82.6% of ‘Alert’ or ‘Monitor’ outcomes in the risk assessment database were similarly classified by the NB classifier.

Table 4 shows that, in comparison to NB, augmentation introduced by the TAN and Multinet classifiers leads to higher precision values for ‘Alert’ and ‘Monitor’ outcomes and for both these outcomes combined. This result indicates that in public health terms TAN and Multinets are better than NB in avoiding false ‘Alert’ and ‘Monitor’ outcomes. This situation is especially the case for the Multinet, with a precision value of 0.846. However, such augmentation leads to lower recall of ‘Alert’ and ‘Monitor’ outcomes, and of both these outcomes combined, in comparison with NB. The Multinet has an especially poor recall (0.177) for ‘Alert’ outcomes.

TAN* and Multinet* take account of the asymmetric data. Comparing TAN and TAN*, Table 4 indicates that for our preferred public health metric recall, the ‘Alert’ outcomes increase from 0.339 to 0.435, the ‘Monitor’ from 0.597 to 0.684 and the combination of these two from 0.649 to 0.754. This improvement is even greater when comparing Multinet and Multinet*. For Multinet* the merged ‘Alert’ and ‘Monitor’ Decision has a recall value of 0.867 indicating that 86.7% of ‘Alert’ or ‘Monitor’ outcomes in the risk assessment database were similarly classified by the Multinet* classifier.

**Fig. 2:**
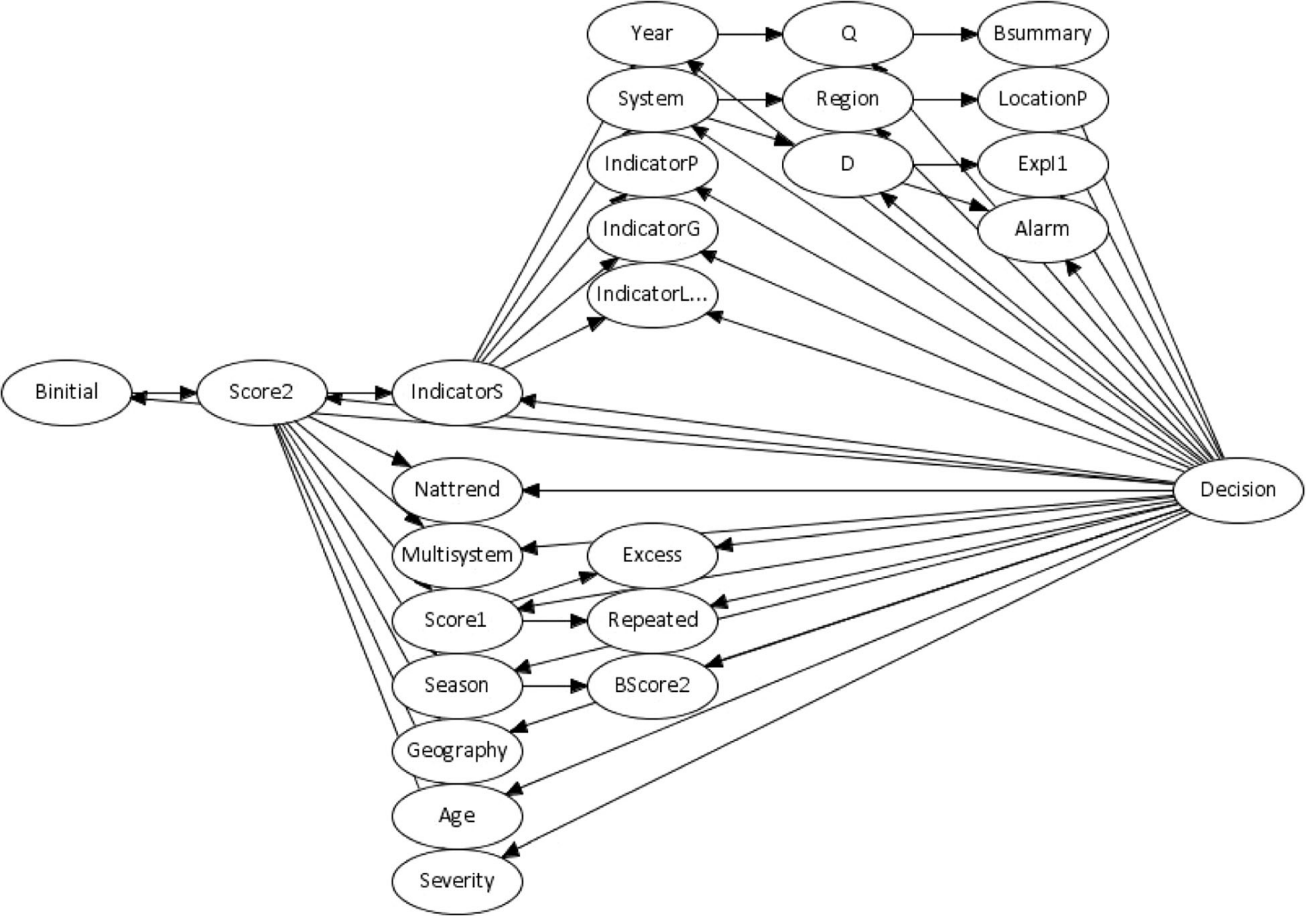
A tree-augmented naïve Bayes network structure induced from details of recorded statistical alarms within the PHE multi-system syndromic surveillance system

The dependency structure of the TAN classification of the variable “Decision”, induced from the recorded statistical alarms in data from the PHE multi-system syndromic surveillance service, is illustrated in Fig. 2. The variable “BInitial” (Is second stage risk assessment initiated?) is chosen as the base of the decision tree because it is the most discriminating attribute in the decision tree construction (Fig. 2). Alternative assignments have minimal effect. The TAN network has 48 links and as expected this structure adds dependency, in the form of directed links, between attributes such as “IndicatorS” and “System” and between “Score1” and “Score2”.

**Fig. 3:**
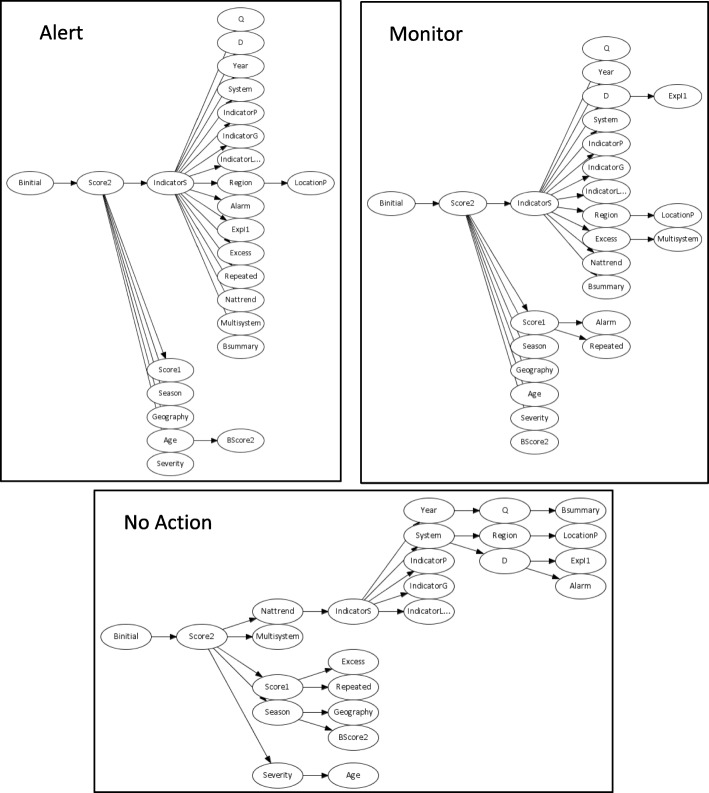
Three components of a multinet classifier, structured as Chow-Liu trees rooted on the “BInitial” variable. Trees corresponding to the ‘Alert’, ‘Monitor’ and ‘No-action’ outcomes of the “Decision” variable are at the top left, top right and bottom of the figure

Figure 3 presents the dependency structure for ‘Alert’, ‘Monitor’, and ‘No-action’ Decisions produced by the Multinet classifier. The results illustrate variation in the dependence of attributes for each decision. For example, for the ‘Alert’ and ‘Monitor’ outcomes there is dependency between “Score2” and “IndicatorS” which is absent for the ‘No-action’ outcome.

In terms of the attributes having most influence upon the Decision outcome, Table 1 includes two measures, evaluated from the risk assessment process. The first measure is mutual information, which expresses how much of the information content of an attribute is shared with the information in the Decision outcome. The second is the *p*-value (calculated using a Pearson χ^2^) which is a measure of statistical significance with respect to a hypothesis that the attribute is independent from the Decision outcome. In this assessment of individual attributes, all of them are significant at a 10% level and only “Q”, “Year” and “Experience” are insignificant at a 1% level. As expected, “BInitial” where a consultant epidemiologist is engaged, and “Score2” the score from stage 2 of the risk assessment process, share most information with the decision outcome. The scores from the second stage of the risk assessment (e.g. Season, Geography, Age, Severity) are also influential on the outcome. The size of the alarm as measured by “Excess”, is the stage 1 attribute that has most influence on the decision.

## Discussion

One important output of our results is that they provide confidence in the current risk assessment process. The attributes of each individual risk assessment shown to be linked to the final decision as the *p*-values in Table 1 were nearly all significant, indicating that the current system is robust and provides consistent results. The five classifiers had relatively high accuracy but this metric was dominated by ‘No-action’ outcomes. From a public heath perspective recall is sometimes more useful, as recall records the proportion of events detected, even if some of these are eventually assessed as false. The NB classifier was shown to have a higher recall for ‘Alert’ and ‘Monitor’ outcomes, in comparison with TAN or Multinet which permit augmentation between attributes. Modified versions of TAN and Multinet, TAN* and Multinet*, were implemented. These modified versions improved their recall.

The NB recall for ‘Alert’ was 0.516 indicating that 51.6% of ‘Alert’ outcomes in the risk assessment database were similarly classified by the NB classifier. This modest recall for ‘Alert’ decisions indicates that additional information is available to the decision maker that is not recorded in the data set. In this respect, it is notable that if the Decisions ‘Alert’ and ‘Monitor’ are combined, then the recall for the NB classifier rises to 0.826. The increased recall when ‘Alert’ and ‘Monitor’ are combined shows that expert decision makers separate ‘Alert’ and ‘Monitor’ outcomes better than machine learned systems and suggests that the information used in the second stage risk assessment is not fully represented in the data set. This result is likely due to public health experience and awareness of wider threats and issues. It is possible that interpretation of this distinction might be helped by data in the textual summary discussed above. In turn, the increased recall when ‘Alert’ and ‘Monitor’ are combined, indicates that the decomposition of the machine learning approach into two or more steps, in accord with the current risk assessment process, may assist with classification.

One potential public health benefit of the findings presented is the ability to use the machine learning results as a decision support system. In the case of PHE’s syndromic surveillance system, an analyst would enter the routine information relating to each statistical event into a computer programme. It would also be possible for some of the statistical event information to be input automatically from the RAMMIE statistical aberration detection system. The system would then process these data, and using the machine learning results from a chosen classifier, present the public health decision probabilities for each outcome. This approach would be presented as Decision probabilities such as 1% ‘Alert’, 3% ‘Monitor’, 96% ‘No-action’. In choosing the most appropriate classifier a strategic decision might be taken to prioritise classifiers which maximise the recall rate for ‘Alert’ outcomes, to enhance the number of alerts correctly returned by the classifier. Hence, based on our results, the NB classifier would be recommended for implementation due to its higher recall values. The advantages of NB have been observed previously in clinical studies and possible explanations are provided elsewhere [24].

Such a decision support system would be particularly useful for the training of new analysts and consultants, providing them with reassurance or otherwise that the decisions made accord with those made in similar circumstances previously. Having a system incorporating multiple classifiers could be particularly useful in this respect. As part of an operator training exercise, high accuracy across all outcomes might be viewed as important, in which case a TAN classifier - which produced the highest accuracy - would be most appropriate.

However, it is imperative to see such a system as a decision support system and not as a decision making tool. Human input is required throughout the risk assessment process and, especially in stage 2, other factors taken into account by the consultant epidemiologist are not recorded by the risk assessment process. Our results highlight the importance of the “BInitial” attribute which relies on human expertise via the engagement of a consultant epidemiologist. Human expertise is also important in the individual components of “Score2”.

The potential uses of these results as a decision support system for syndromic surveillance, fit into a wider UK ambition to make machine learning and artificial intelligence techniques more accessible and to deliver complex data into the hands of specialist groups such as health professionals [25]. This goal is particularly relevant in relation to medicine and health because of a growing complexity in the provision of care, coupled with a rapid move to digital information collection. Optimization of healthcare informatics is an emerging issue and leverage of new data sources pertaining to syndromic surveillance is a priority. Recently, there have been innovative uses for machine learning methods in relation to diagnostics and prognostication for human and veterinary medicine [26, 27], but applications in healthcare surveillance are rare [28, 29].

This analysis could be extended, and one improvement could be gained by obtaining information from the textual summary present on each record. The textual summary was present in approximately 30% of events, but replaced by a simple binary (yes/no) value for this analysis. Natural language processing may be an approach to obtain further information from this field which could then be fed into machine learning (e.g. [29]). A further area for development is that the NB classifiers used in this paper assume that all attributes are conditionally independent given the Decision outcome. If this condition is not met, the corresponding classification may become biased. This bias issue was overcome using TAN and Multinets which have network augmentation, which are additional links representing dependency between attributes. An extension to this research would be to implement an alternative approach that avoids augmentation but addresses the issue of attributes not being conditionally independent. This alternative is called feature selection and classifies using a subset of the attributes that do not include strong dependencies (e.g. [15, 30]). A notable challenge in such an approach is the identification of an appropriate subset of attributes.

The evaluation of classifier performance and the learning process itself, relies on the risk assessment database. This manually labelled database is treated as established truth, but the reality is that actual truth is rarely available and difficult to separate from the effect of any actions. Put simply, the outcomes in the risk assessment database are the outcomes of the risk assessment process but may not be correct in a public health sense. This means that we do not know for certain whether a statistical event should have been labelled as an Alert. Furthermore, we acknowledge that the labelling process includes uncertainty, for example, in situations that are equivocal, as well as some inter-operator variability. Both of these issues will degrade measured classifier performance [31].

The classifiers used in this paper assume that the relationships in these data did not change over time. In reality, our experience is that decision makers learn from experience. Hence, any decision support system based on machine learning would need to be future-proofed by linking the learning step to a data stream with a fading memory to ensure that the machine learning would be regularly updated from the most recent data. In addition, syndromic systems need to cope with emerging threats for which there may be no precedent in the training data set, thus again highlighting the importance of human input throughout the process.

## Conclusions

This paper has successfully explored the use of machine learning methods to assist public health decision making in an operational multi-system syndromic surveillance. The attributes of each individual risk assessment were shown to be linked to the final decision providing confidence in the current process. The NB classifier was shown to have a higher recall for ‘Alert’ and ‘Monitor’ outcomes in comparison to TAN or Multinet. The NB recall for ‘Alert’ was 0.516 indicating that 51.6% of ‘Alert’ outcomes in the risk assessment database were similarly classified by the NB classifier. When the Decisions ‘Alert’ and ‘Monitor’ are combined, then the recall for the NB classifier rises to 0.826. The combination of ‘Alert’ and ‘Monitor’ indicates that expert decision makers separate ‘Alert’ and ‘Monitor’ outcomes better than machine learned systems and suggests that the information used in the second stage risk assessment is not fully represented in the data set. The machine learning techniques could be developed into a decision support system for risk assessment within syndromic surveillance. In such a system, the analyst would input the risk assessment data, and be presented with a set of Decision probabilities such as ‘Alert’ 1%, ‘Monitor’ 25%, ‘No-action’ 74%. This system would be particularly useful for the training of new analysts and consultants, providing reassurance or otherwise that the decisions made accord with those in the past. Should such a system be implemented, a NB classifier would be a valuable starting point as this would maximise the number of ‘Alerts’ identified. It is also fast, robust and relatively insensitive to missing values. Within such a system we highlight the importance of introducing a fading memory so that the classifier is regularly updated to take account of new data and the fact that decision makers learn from experience. Finally, it is essential to see such a system as a means to support decision making as our results highlight the importance of specialist human input with public health knowledge throughout the process.

## Abbreviations

EDSSS: Emergency Department syndromic surveillance system
GP: General Practitioners
GPIHSS: In-hours general practitioner syndromic surveillance system
GPOOHSS: Out-of-hours hours general practitioner syndromic surveillance system
MCC: Matthews correlation coefficient
Multinet: Bayesian Multinets
Multinet*: Multinet with the minority class (Decision = ‘Alert’) oversampled by ~ 1000
NB: Naïve Bayes
NHS: National Health Service
NHS111: National Health Service telephone advice system
NHS24: National Health Service telephone advice system
PHE: Public Health England
RAMMIE: Rising activity, multi-level mixed effects, indicator emphasis method for syndromic surveillance
TAN: Tree-augmented Naïve Bayes
TAN*: TAN with outcome classed as ‘Alert’ if the ‘Alert’ probability was ≥0.2. If not, classified as ‘Monitor’ if the ‘Monitor’ probability was ≥0.2
